# Ubiquitin-positive astrogliopathy clinically mimicking Parkinson’s disease

**DOI:** 10.1186/s40478-022-01464-y

**Published:** 2022-11-14

**Authors:** Meaghan Morris, Abhay Moghekar, Haidan Guo, Olga Pletnikova, Javier Redding-Ochoa, Marilyn Albert, Susan M. Resnick, Liam Chen

**Affiliations:** 1 Department of Pathology, Johns Hopkins University, Baltimore, MD USA; 2 Department of Neurology, Johns Hopkins University, Baltimore, MD USA; 3 Department of Pathology and Anatomical Sciences, University at Buffalo, Buffalo, NY USA; 4 Laboratory of Behavioral Neuroscience, National Institute on Aging, Baltimore, MD USA; 5 Department of Laboratory Medicine and Pathology, University of Minnesota, Minneapolis, MN USA

**Keywords:** Ubiquitin, Astrogliopathy, Parkinsonism, Neurodegeneration

## Abstract

Several neurodegenerative pathologies can clinically mimic Parkinson’s disease, including neurodegenerative diseases with glial pathology. However, the glial aggregates are typically composed of known pathogenic proteins and are associated with prominent neuronal loss in the substantia nigra. Here we present an unusual case of a 91-year-old man with a clinical diagnosis of Parkinson’s disease, but whose autopsy findings showed a ubiquitin-positive astrogliopathy without significant neuronal loss in the substantia nigra. These glial aggregates affected the basal ganglia, cortex, and cerebellum, and were negative for tau, alpha-synuclein, TDP-43, FUS, and p62. This case is a rare example of an unknown glial neurodegenerative pathology mimicking Parkinson’s disease without significant loss of nigral dopaminergic neurons.

## Introduction

A variety of neurodegenerative pathologies can cause clinical Parkinsonism, some of which closely mimic Parkinson’s disease. The majority of these neurodegenerative diseases cause prominent loss of pigmented dopaminergic neurons in the substantia nigra, which is thought to underlie many of the clinical motor impairments. While several clinical mimics of Parkinson’s disease can show prominent glial pathology, often the aggregates are comprised of known pathogenic proteins such as tau and alpha-synuclein. Herein we present an unusual case of a man with clinical symptoms mimicking Parkinson’s disease but whose autopsy findings showed a ubiquitin-positive astrogliopathy and no significant loss of dopaminergic neurons in the substantia nigra.

## Case presentation

The patient was a 91 year-old man with a long-standing history of dopamine-responsive Parkinsonism. He presented with dysphagia approximately 14 years prior to death. He then developed an asymmetric resting tremor, reduced arm swing, stooped posture, and a shuffling gait which responded to Carbidopa-Levodopa therapy. He was clinically diagnosed with Parkinson’s disease 13 years prior to his death. His motor symptoms gradually worsened, he suffered repeated falls, and eventually developed orthostatic hypotension.

As part of his participation in the Baltimore Longitudinal Study of Aging (BLSA) and the Johns Hopkins Alzheimer’s Disease Research Center (JHADRC), he underwent repeated neuroimaging and cognitive testing. The last magnetic resonance imaging (MRI) study, approximately 5 years prior to death, showed only moderate age-related parenchymal volume loss. Based on clinical and cognitive evaluations at serial BLSA visits, the participant was judged to be cognitively normal (based on standard diagnostic consensus criteria) through the final visit, which occurred approximately 2 years prior to death. He had no known history of toxin exposure and no history of psychiatric symptoms or anti-psychotic therapy. There was no family history of movement disorders or dementia. As part of the JHADRC protocol, the patient underwent an autopsy restricted to the brain after death.

At autopsy, the gross brain weight was 1450 g. The left hemisphere, brainstem, and cerebellum were sectioned fresh, while the right hemisphere was fixed in formalin for other studies. No gross photographs were obtained. There was no significant cortical atrophy noted, though there was mild *ex vacuo* hydrocephalus of the lateral ventricle. There was no evidence of atrophy or discoloration of the basal ganglia. Diagnostic sections were taken from fresh tissue and the remaining tissue was frozen and banked. On gross examination, the locus ceruleus and substantia nigra were grossly well pigmented and histologic examination showed an age-appropriate population of pigmented neurons. Hematoxylin and eosin (H&E) staining showed a mildly prominent Bergmann glial cell layer in the cerebellar cortex and neurofibrillary tangles in the hippocampus, entorhinal cortex, and locus ceruleus. There was no overt neuronal loss, atrophy, or glial inclusions on H&E staining of the basal ganglia. Modified Bielschowsky silver staining showed a primary age-related tauopathy (PART) (Braak IV) [1], with no evidence of amyloid deposition by silver staining or amyloid immunostaining (6 F/3D, DAKO, #M0872, 1:100). There were no Lewy bodies present on hematoxylin and eosin staining, and no accumulation of alpha-synuclein in neurons or glia by immunohistochemistry (42, BD Transduction Biosciences, #610,786, 1:100). While there was mild arteriolosclerosis, the was no evidence of ischemic vascular pathology excluding vascular Parkinsonism.

**Fig. 1:**
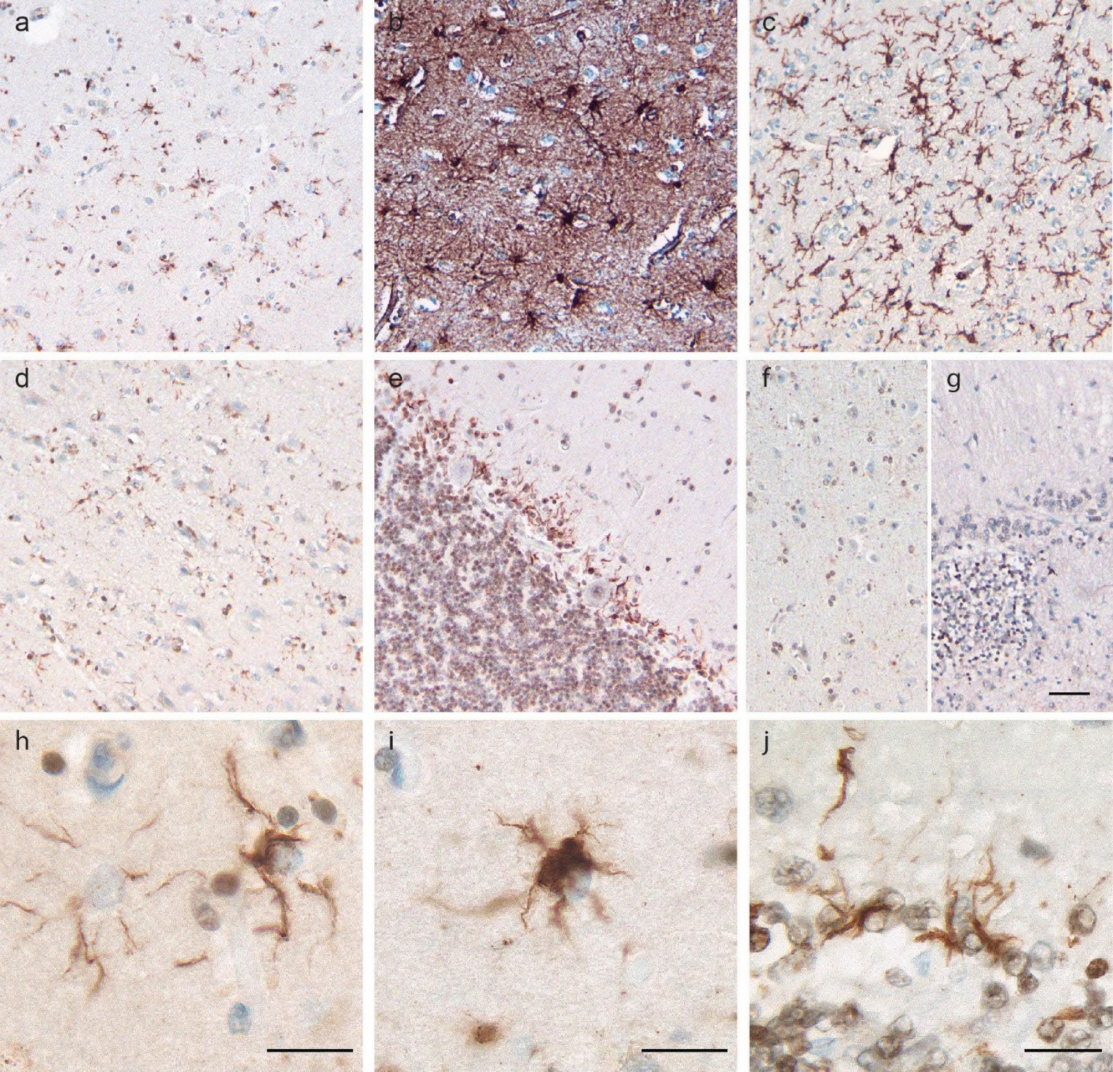
Pathologic Findings in Ubiquitin-Positive Astrogliopathy. Ubiquitin-positive glial inclusions are most prominent in the basal ganglia (a), associated with GFAP-positive gliosis (b) and increased Iba-1-positive activated microglia (c). Ubiquitin-positive glial inclusions also affected cortex (d), and the Bergmann glial layer of the cerebellar cortex (e). Control cases with primary age-related tauopathy do not show ubiquitin-positive glial inclusions in the mesial temporal cortex (f) or associated with Bergmann gliosis over a chronic infarct in the cerebellum (g). Ubiquitin-positive glial inclusions are typically fine linear aggregates in glial processes (h) with scattered glia also showing dense aggregates in the cell body (i). Linear accumulation in the glial processes of the cerebellum is prominent in the Bergmann glial cell layer (j). Scale bars are 50 μm (g) and 20 μm (h-j). Scale bar in g applies to a-g

Ubiquitin immunostaining (Ub-1, Chemicon, #MAB1510, 1:2000) showed diffuse glial ubiquitin-positive inclusions which were most prominent in the basal ganglia (Fig. 1a). Areas of higher density of glial inclusions in the basal ganglia were associated with gliosis by GFAP immunostaining (DAKO, #Z0334, 1:500) and an increased density of activated microglia by Iba-1 staining (Wako, #019-19741, 1:800) (Fig. 1b-c). The next most densely affected areas were the cerebral cortex and the Bergmann glial cell layer in the cerebellar cortex (Fig. 1d-e). A control case with PART showed no evidence of ubiquitin-positive glial pathology in the mesial temporal cortex (Fig. 1f). Immunostaining of multiple cases of PART with either no cerebellar pathology or Bergmann gliosis associated with cerebellar infarcts showed no evidence of ubiquitin-positive glial inclusions (Fig. 1g). In the cortex, all layers were affected, though the deeper layers tended to have more dense glial pathology compared to the superficial layers. There was no subpial, subependymal, or perivascular arrangement of the inclusions.

**Fig. 2:**
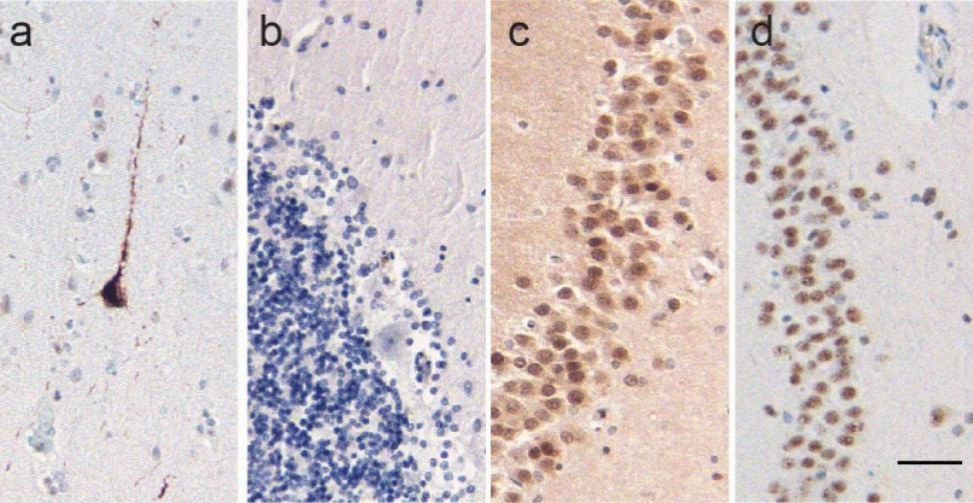
Other Pathologic Findings. Phospho-tau immunostaining (AT8) shows primary age-related tauopathy involving neurons (a), but does not show aggregates in glia. Immunostaining for p62 is negative in the glial aggregates (b). Immunostaining in the hippocampal dentate gyrus shows no evidence of FUS (c) or TDP-43 (d) aggregation. Scale bar is 50 μm (d) and applies to a-d

The glial aggregates were typically fine linear inclusions in glial processes (Fig. 1h), though there were scattered glia with more dense cell body accumulation (Fig. 1i) which were most common in the basal ganglia. The cerebellar glial inclusions were distinctive, occurring solely in the cerebellar Bergmann glial cell layer and showing a roughly radial orientation toward the surface of the cerebellar cortex (Fig. 1j). The ubiquitin-positive glial inclusions spared the white matter, thalamus, and brainstem, including the substantia nigra. No definite pathologic inclusions were identified in neurons, aside from those associated with PART. Phosphorylated tau immunostaining (Fig. 2a) (AT8, Research Diagnostics, Inc. #MN1020B, 1:50) confirmed PART but was negative in the glial aggregates and did not show age-related tau astrogliopathy (ARTAG) [3]. Another commonly used phosphorylated tau antibody (PHF1, Peter Davies lab, 1:200) showed a similar staining pattern (data not shown). p62 immunostaining (3, BD Transduction Laboratories, #610,833, 1:50), performed in multiple brain regions in this case, also highlighted neurofibrillary tangles but was negative in the glial cytoplasmic inclusions (Fig. 2b). FUS (Fig. 2c) (ag2150, ProteinTech, #11570-1-AP, 1:50) and TDP-43 (Fig. 2d) (ProteinTech, #10782-2-AP, 1:2000) were negative in the glial inclusions and showed no evidence of neuronal pathology.

## Discussion and conclusions

This case represents a highly unusual neurodegenerative process resulting in clinical Parkinsonism without significant loss of dopaminergic neurons. In general, neurodegenerative causes of Parkinsonism are unified by the prominent loss of dopaminergic neurons in the substantia nigra [2], which accounts for many of the dopamine-responsive motor impairments seen in Parkinson’s disease. Even Parkinson’s disease with strong genetic contributors, which can have variable pathologic aggregates, usually shows loss of dopaminergic neurons in the substantia nigra when examined pathologically [6]. In this case, the patient had dopamine responsive motor symptoms, but the major concentration of pathology was located in the basal ganglia which is the target of dopaminergic projections from the substantia nigra.

Clinical Parkinsonism may be caused by environmental or therapeutic exposures without loss of dopaminergic neurons in the substantia nigra, however, these have not been associated with protein aggregation. Manganese toxicity can cause Parkinsonism associated with loss of neurons and gliosis in the globus pallidus with no significant neuronal loss in the substantia nigra pars compacta [5]. By contrast, Parkinsonism caused by anti-psychotic medication often has no pathologic changes at all [2]. While these examples highlight the potential pathologic diversity underlying clinical Parkinsonism, they have not been associated with degenerative Parkinsonism with protein aggregation as was present in this case.

The pathogenic protein in the glial aggregates in this case is unknown. Glial pathology is well documented in several neurodegenerative pathologies underlying atypical Parkinsonism. However, the glial aggregates are usually comprised of known pathogenic proteins, such as alpha-synuclein, tau, or TDP-43 [2], which were all negative in this case. Interestingly, p62 was also negative in the glial aggregates, with the internal control of p62-positive neurofibrillary tangles associated with PART. Ubiquitin-positive aggregates in most neurodegenerative diseases are typically co-positive with p62. However, ubiquitin immunostaining has been reported to be more sensitive for specific types of pathologic aggregates, including glial cytoplasmic inclusions in multiple system atrophy, neuritic tau and synuclein aggregates, and some inclusions in FTLD-U [4]. This raises the possibility that either these inclusions are not as compact as those typically labeled by p62, or that this is one of the rare inclusion types for which ubiquitin immunostaining is more sensitive.

The pathologic classification of this case is also unclear. The frontotemporal lobar degeneration with ubiquitin-positive inclusions (FTLD-U) pathologic classification includes cases with unknown ubiquitin-positive aggregates. However, it is not clear that this case represents FTLD-U given the lack of frontotemporal atrophy, cognitive impairment, and behavioral changes. If additional cases were identified of this unusual neurodegenerative pathology, future studies could expand upon the clinical and pathologic phenotype, as well as investigate the protein composition of these unusual aggregates.

## Data Availability

Not applicable.

## Abbreviations

ARTAG: age-related tau astrogliopathy
BLSA: Baltimore Longitudinal Study of Aging
FTLD-U: frontotemporal lobar degeneration with ubiquitin-positive inclusions
JHADRC: Johns Hopkins Alzheimer’s Disease Research Center
MRI: magnetic resonance imaging
PART: primary age-related tauopathy
